# Textural and Sensory Features Changes of Gluten Free Muffins Based on Rice Sourdough Fermented with *Lactobacillus spicheri* DSM 15429

**DOI:** 10.3390/foods9030363

**Published:** 2020-03-20

**Authors:** Maria Simona Chiş, Adriana Păucean, Simona Maria Man, Vlad Mureşan, Sonia Ancuţa Socaci, Anamaria Pop, Laura Stan, Bogdan Rusu, Sevastiţa Muste

**Affiliations:** 1 Department of Food Engineering, Faculty of Food Science and Technology, University of Agricultural Sciences and Veterinary Medicine of Cluj-Napoca, 3–5 Mănăştur Street, 400372 Cluj-Napoca, Romania; simona.chis@usamvcluj.ro (M.S.C.); simona.man@usamvcluj.ro (S.M.M.); vlad.mureşan@usamvcluj.ro (V.M.); anamaria.pop@usamvcluj.ro (A.P.); sevastita.muste@usamvcluj.ro (S.M.); 2 Department of Food Science, Faculty of Food Science and Technology, University of Agricultural Sciences and Veterinary Medicine of Cluj-Napoca, 3–5 Mănăştur Street, 400372 Cluj-Napoca, Romania; sonia.socaci@usamvcluj.ro (S.A.S.); laurastan@usamvcluj.ro (L.S.); 3 Institute of Life Sciences, University of Agricultural Sciences and Veterinary Medicine Cluj-Napoca, 3–5 Calea Mănăştur, 400372 Cluj-Napoca, Romania; rusubogdan94@gmail.com

**Keywords:** *Lactobacillus spicheri* DSM 15429, texture, sensory, volatiles

## Abstract

Gluten free products available on the market have a low textural quality associated with high crumbly structure, low-flavor, aroma, poor mouthfeel, less appearance, in comparison with the conventional final baked products. The aim of this study was to assess the influence of rice sourdough fermented with *Lactobacillus spicheri* DSM 15429 strain on textural, volatile profile, and sensorial properties of gluten free muffins in order to obtain baked goods with improved quality characteristics. *Lactobacillus spicheri* is a novel strain isolated from industrial rice sourdough but unexploited for bakery products manufacturing. The results showed that *Lactobacillus spicheri* DSM 15429 was able to growth in the rice flour influencing the texture and the volatile profile of gluten free muffins as well as their sensory characteristics. Both, textural parameters and volatiles recorded significant differences comparing to muffins obtained with a spontaneously fermented rice sourdough. Hardness and cohesiveness decreased while springiness and resilience of gluten free muffins improved their values. The volatile profile of gluten free muffins was significantly improved by utilization of the rice sourdough fermented with *Lactobacilus spicheri* DSM 15429. 3-methylbutanal, 2-methylbutanal, acetophenone and limonene were the main volatile derivatives responsible for aroma and odor scores of sensory analysis.

## 1. Introduction

In recent years, the prevalence of celiac disease has increased and recently, a new syndrome called non-celiac gluten sensitivity (NCGS) has been identified and confirmed [1,2]. The disorders related to the presence of gluten such as celiac disease, wheat allergy and NCGS, are continuously increasing reaching up to 5% of total global prevalence [3].

Generally, gluten free baked products are characterized by reduced textural and sensorial properties compared to their gluten-containing counterparts [4]. Because of the flours employed, gluten free biscuits may be harder, may present a dry and sandy mouthfeel and an unpleasant appearance, taste, or color [5]. In fact, the gluten free products available on the market have a low textural quality associated with high crumbly structure, low-flavor, aroma, poor mouthfeel, less appearance, in comparison with the conventional final baked products [1,6,7,8,9,10]. Beside the poor technological quality such as crumbling crumb, low volume, gluten free products have also nutritional problems being rich in fat and poor in protein [11].

Nowadays, there is a renewed interest in gluten free cereal fermentation with lactic acid bacteria which is correlated with a greater amount of bioactive compounds and with benefits on the texture, shelf-life, food preservation and sensorial features of the final baked goods, opening new research frontiers in food manufacture [12,13].

One of the most used gluten free cereal is rice, a leading cereal crop, which is rich in protein, energy and minerals, but, unfortunately, does not fulfill the daily nutritional human requirements for micronutrients [14] due to the milling process. Rice flour is largely employed in gluten free products due to its bland and neutral taste and usually is combined with more tasteful flours like buckwheat flour [5].

The use of gluten free rice flour in food manufacture is restricted by the final quality of sensorial features and due to the low baking quality. Fermentation with LAB could be a possibility to improve this technological and sensorial issues [15,16,17]. Also, Stantiall and Serventi (2018) [6] reported that the products manufactured with rice flour offer to the consumers’ poor specific volume and density, having inferior viscoelastic properties.

Thus, there is a necessity to improve gluten free products quality through new technological processes, and one of it could be sourdough fermentation with lactic acid bacteria (LAB). Rice is a staple food for almost 50% of the world’s population, being an important source of nutrients that could be successfully used as a substrate for the growth of LAB during fermentation process [15,17,18].

LAB are microorganisms with an essential role in sourdough production being involved in the production of the aroma compounds, the bioavailability of minerals, the amount of phenolic compounds and the protein degradation [19]. Overall, fermentation by LAB is considered a tool that might improve the rheology, sensory, shelf life and nutritional characteristics of the food matrix [3]. Also, Gobbetti et al., (2019) [20] reported the incontrovertible key role of the LAB in improving the texture, sensory and nutritional features of the raw matrix.

The most representative genus of LAB is *Lactobacillus*, *Leuconostoc, and Lactococcus* [21], and many studies are being published lately about genome sequences of *Lactobacillus* species. A new strain of the genus *Lactobacillus*, *Lactobacillus spicheri* strain was first described and isolated in 2004, from industrial rice sourdoughs by Meroth et al., (2004) [22]. In another study, by Vogelmamm et al., (2009) [15], it was shown that *Lactobacillus spicheri* DSM 15429 strain is able to grow in different vegetable matrix such as cassava, rye and amaranth. *Lactobacillus spicheri* G2 strain was isolated by Gautant and Sharma (2005) [23] from gundruk (a vegetable product from India and Nepal) being considered a probiotic strain which could be used in the production of functional foods. Traditional fermented food made from rice through lactic acid bacteria fermentation are most common in Thailand, China, India and belong to the genus *Lactobacillus* [24].

The aim of this study was to assess the influence of rice sourdough fermented with *Lactobacillus spicheri* DSM 15429 strain on textural, volatile profile and sensorial properties of gluten free muffins in order to obtain baked goods with improved quality characteristics.

## 2. Materials and Methods

### 2.1. Standards, Reagents, and Raw Materials

*Lactobacillus spicheri* DSM 15429 (Lsp) and Man Rogosa Sharpe (MRS) medium were acquired in lyophilized form from Leibniz Institute-German Collection of Microorganism and Cell Cultures (Germany, Brunswick) and from Merck (Darmstadt, Germany), respectively.

Lactic acid was acquired from Fluka (Germany, Munich). The mix standard solutions (ICP Multielement Standard solution IV CertiPUR) used for calibration curve, were achieved from Merk (Darmstadt, Germany). All reagents were of analytical grade. Analytical reagents and chemicals were purchased from Sigma Aldrich (St. Louis, MO, USA).

Rice integral flour without gluten (originated from Germany) and the other raw materials were purchased from the local market, from specialized stores in Romania.

### 2.2. Chemical Composition of Rice Integral Gluten Free Flour

Chemical composition of rice flour (moisture, ash, protein, lipids) was determined according to AACC approved methods 45-15 A, 08-01, 46-11A, 30-10.01, [25], as showed in Table 1. Carbohydrates were calculated according to method described by Rizzelo et al., (2017) [26].

### 2.3. Microbial Starter Culture Preparation

*Lactobacillus spicheri* DSM 15429 (Lsp) was grown in MRS medium at 30 °C, during 48 h, according to Meroth et al., (2004) [22]. Sourdough was prepared as described by [8] as follows: cells were harvested by centrifugation (2300× *g*, 10 min, 4 °C) using Eppendorf 5804 centrifuge (Germany, Hamburg), washed, re-suspended in sterile tap water and added into the sourdough so that an initial cell count of 10^7^ cfu/g sourdough was reached. Sourdough was manufactured by mixing the rice flour: water (1:1, *w*:*v*) obtaining a final dough yield of 200. The fermentation was made during 24 h, at 30 °C on an orbital shaker (Heidolph Unimax 1010 Inkubator 1000, Germany) with 170 rpm. During fermentation, samples were withdrawn at different time intervals (0, 4, 8, 12, 24 h), and coded as showed in Table 2.

As a negative control, the sourdough without inoculum of Lsp (OR) was prepared and fermented under the same conditions as the sourdough with Lsp (SP).

### 2.4. Lactobacillus Spicheri DSM 15429 Adaptability in Rice Sourdough (Cell Counts, pH, Total Titrable Acidity, and Lactic Acid Amount)

Cell counts, pH and TTA (total titrable acidity) values of SP and OR sourdoughs were analyzed according to the methods described by Chiș et al., (2018) [27]. Briefly, Lsp cells were spread on MRS agar media and incubated at 30 °C for 48 h. 5 mL of each sourdough sample was suspended in 45 mL of sterile sodium chloride (0.85% *w*/*v*) and homogenized by vortexing. One milliliter was used for serial dilutions and also for plating with MRS agar. The plates were incubated at 30 °C during 48 h. For Lsp counting, serial dilution technique and pour plate method were used.

The pH values were determined by using a WTW pH-meter (Hanna Instruments, Vöhringen, Germany) and TTA values were obtained by mixing 10 g of SP respectively OR sourdoughs with 90 mL distilled water. The solution was neutralized with sodium hydroxide (NaOH 0.1 N) using phenolphthalein as indicator, until the pH reached a value of 8.3 units. The TTA values were expressed as the amount of NaOH (mL).

Lactic acid was analyzed by high-performance liquid chromatography analysis HPLC-UV detection (Agilent Technologies 1200 Series, Japan, Kyoto) for organic acids, according to the method previously published by Păucean et al., (2013) [28]. A standard curve of lactic acid (y = 16.93x + 149.26, R^2^ = 0.997) was used to establish the concentration of lactic acid (mmoL/L) according to the method described by Chiș et al., (2018) [27].

### 2.5. Muffins Preparation

The SS and SP sourdoughs fermented at 0, 12, and 24 h were used in muffins manufacture, as showed in Table 3. Firstly, the rice integral flour was subjected to a hydrothermal treatment, which is considered as a bakery improver, as described by Bourekoua et al., (2016) [29]. After that, the rice treated flour, corn starch, baking powder, inulin, buckwheat flour, egg yolk, and SP or OR sourdoughs were mixed for two minutes in a mixer (KitchenAid^®^ Precise Heat Mixing Bowl., Ohio, OH, USA), at medium speed. The egg white was whipped and homogenized with maple syrup and added slowly in the first mixture. Fifty grams of batter was used to fill the specific baking trays. An electric oven (Zanolli, Verona, Italy) was used for baking the muffins at 200 °C for 10 min and further at 180 °C for 6 min, as presented in our previous work [30]. After baking, the muffins were cooled down at pilot station bakery, packed in vacuum bags at 850 mbar (Multivac C 200, Multivac, Wolfertschwenden, Germany) and coded as showed in Table 4.

### 2.6. Texture Profile Analysis for Muffins Samples

The texture profile analysis of the muffins was performed by using CT 3 Texture Analyzer (Brookfield Engineering Labs, Middleboro, MA, USA) and according to the method described by Goswami et al., (2015) [31], with slight modifications. The CT 3 Texture Analyzer was equipped with a 10 kg load cell and the TA25/1000 probe (50.8 mm diameter AOAC Standard Clear Acrylic 23 g, 20 mm length). The muffin samples were subjected to a double compression test under the following conditions: 50% target deformation, 1 mm s−1 test and post-test speed, 5 g trigger load, and 5 s recovery time. Hardness, total work, resilience, springiness, cohesiveness, gumminess and chewiness of the crumb were used as indicators for the texture profile analysis.

Each muffin sample was cut manually with a stainless steel knife, crumb samples of 25 × 25 × 25 mm (l × w × h) being obtained. In order to avoid crumb drying, the samples were tested immediately after preparation. The specific texture parameters were performed using Texture Pro CT V1.6 soft- ware (Brookfield Engineering Labs, Middleboro, MA, USA).

### 2.7. Extraction and Analysis of Volatile Compounds by ITEX/GS-MS

The extraction of volatile compounds was made according to the method described by Socaci et al., (2014) [32]. In brief, five grams of each sourdough sample was placed in a 20 mL headspace vial, incubated for 20 min at 60 °C, under continuous agitation. The polymer fiber microtrap (ITEX-2TRAPTXTA, Tenax TA 80/100 mesh) was used to absorb continuously (30 strokes) the volatile compounds with a headspace syringe (CombiPAL AOC-5000 autosampler, CTC Analytics). The volatile compounds were then thermally desorbed directly into the GS-MS injector.

The analysis of volatile compounds was carried out on a gas chromatograph-mass spectrometer GC-MS model QP-2010 (Shimadzu Scientific Instruments, Kyoto, Japan), using the method described by Socaci et al., (2014) [32]. The separation of volatile compounds was performed on a Zebron ZB-5 ms capillary column of 30 m × 0.25 mm i.d and 0.25 mm film thickness. The carrier gas was helium, 1 mL/min, split ratio 5:1, injector temperature 250 °C. The temperature program of the column oven was: 35 °C (hold for 5 min) to 155 °C at 7 °C/min to 260 °C at 10 °C/min and hold for 5 min. The MS detection was performed on a quadrupole mass spectrometer operating in full scan (40–450 *m*/*z*) electron impact (EI) at ionization energy of 70 eV. The spectra of reference compounds from NIST27 and NIST147 mass spectra libraries were used in order to identify the separated compounds and verified by with retention indices drawn from [33,34].

### 2.8. Sensory Evaluation

The sensory characteristics of muffins such as appearance, color, texture, taste, flavor, texture, and overall acceptability were analyzed by 37 panelists (65% female and 35% male, range 19–63 years), according to the method described by Coda et al., (2010) [35], with slight modifications, according to Chiș et al., (2020) [36]. A nine hedonic scale was used by the assessors to evaluate the sensory features, ranging from 1–4 which represent negative sensations, 5 was neither like nor dislike, to 6–9 which represent positive sensations, 9 meaning like very much.

### 2.9. Statistical Analysis

Data were analyzed using Duncan multiple comparison test by using SPSS version 19 software (IBM Corp., Armonk, NY, USA). Significant differences between OR and SP sourdoughs and the final products were indicated by different small letters. The results of three independent (*n* = 3) assays performed with replicates each were expressed as means ± standard deviations. Pearson correlation was also used to correlate Lsp cellular growth with lactic acid and TTA amounts.

## 3. Results and Discussions

### 3.1. Lactobacillus Spicheri DSM 15429 Adaptability in Rice Sourdough (Cell Counts, pH, TTA, and Lactic Acid Amount)

*Lactobacillus spicheri* is defined by Petel et al., (2017) [37] as a heterofermentative LAB, being characterized by the conversion on the hexoses into lactic and acetic acids, ethanol and CO_2_. In the present study, due to the good adaptability of Lsp and to the nutritious composition of rice integral flour represented by carbohydrates, proteins, minerals, the development of Lsp strain was successful.

**Figure 1:**
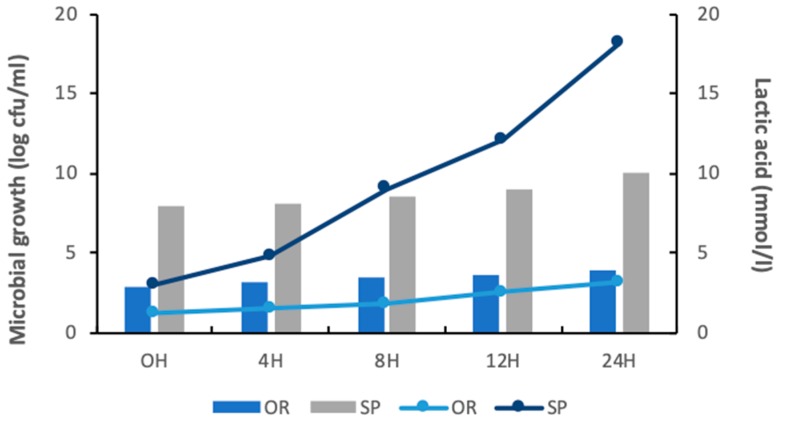
Microbial growth (log cfu/mL) and formation of lactic acid (mmoL/L) in spontaneously fermented sourdough (OR) and sourdough fermented with *Lactobacillus spicheri* DSMZ 15429 (SP) during 24 h of fermentation.

The growth of Lsp was observed during 24 h of fermentation at 30 °C and reached a final value of 9.9 log cfu/g sourdough, as showed in Figure 1. The cells of *Lactobacillus spicheri* were rod-shaped, having in MRS agar white colonies, circular with a smooth surface and a diameter of 0.5 to 1 mm. These morphological characteristics were used to identify Lsp cells according Meroth et al., (2004) [22] and also, Lsp cells were cultivated on MRS agar and the morphology of the colonies was used as a model for comparing.

In the plates with non-inoculated sourdough (OR) a mixed microbiota composed by long Gram (+) or Gram (−) bacilli and diplococci was observed using the Gram coloration method, that was not able to ensure a good acidification rate, during 24 h of fermentation, reaching a final value of only 3.9 log cfu /g sourdough.

**Figure 2:**
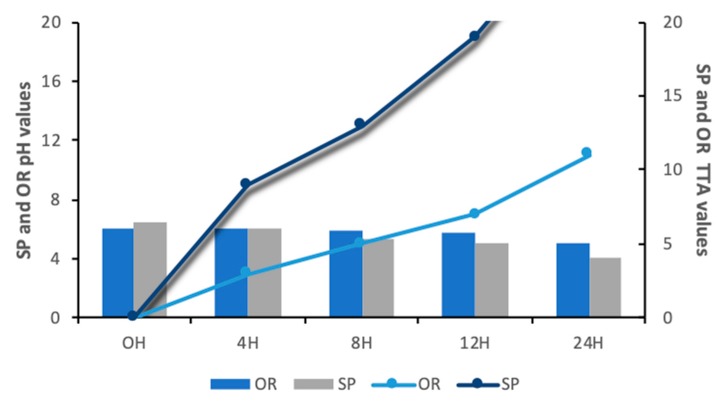
The evolution of pH and total titrable acidity (TTA) of both spontaneously fermented sourdough (OR) and of sourdough fermented with *Lactobacillus spicheri* DSMZ 15429 (SP) during 24 h of fermentation.

After 24 h of fermentation, the SP pH value was 4.1, compared to the OR pH, the value of which was 5.0. Regarding the TTA value, the sourdough SP, after 24 h of fermentation had a value of 17 (mL NaOH) compared to the OR TTA value, which was only 11 (mL NaOH), as showed in Figure 2. The pH and TTA changes during OR fermentation are due to the good adaptability of wild flour rice microflora and depends on the ecological factors being different from a region to another [37].

The SP lactic acid content after 24 h of fermentation was 4.8 fold higher than in OR sourdough, showing that the Lsp was able to replace the rice flour microbiota, reaching a value of 15 mmoL/L, compared to the non-inoculated sourdough which reached only a value of 3.1 mmoL/L lactic acid.

The ability of LAB to produce bioactive molecules such as lactic acid, has been confirmed by [38,39,40]. Lactic acid is produced from carbohydrates through fermentation and represent a key factor with a big influence on the flavor of the final baked goods, being considered as a flavor-active product according to [41,42,43].

The growth of Lsp could be corelated with a lower amount of pH, higher amount of lactic acid and with the acidification of the raw matrix. High Pearsons values (0.98 and 0.94) indicate a strong relationship between the growth of Lsp and the lactic acid and between the growth of Lsp and TTA values. These findings confirm the previously published findings, [38], who reported that lactic acid bacteria such as *Lactobacillus plantarum* CRL 778 is able to growth in quinoa sourdough influencing the fermentation process through the decrease of the pH and the increase of lactic and acetic acids. Also, [44] showed that *Lactobacillus sanfranciscensis* LS40 and LS41 *and Lactobacillus plantarum* CF1 were able to growth in a mixture of rice, buckwheat, and maize flour, influencing the acidification rate of the sourdough due to the lactic acid formation. In a recent study, [45] showed that different strains of *Lactobacillus* such as *Lactobacillus brevis, Lactobacillus paralimentarius* could be used in the manufacture of pearl millet gluten free sourdough influencing the pH and TTA values. Moreover, Nami et al., (2019) [45] reported that the pearl millet sourdough fermented with lactic acid bacteria could be used as an improver for texture and the sensory properties of the final baked good.

By considering that in this study the chemical composition of the raw matrix was the same, the temperature and the fermentation time were kept constant, only factors like the interaction between LAB and spontaneous biota of the matrix could influence the fermentation process, according to [15].

### 3.2. Texture Profile Analysis

Results of the texture profile of muffins made with SP and OR sourdough at different fermentation times are listed in Table 5. Hardness is defined as the peak force of the first compression cycle meanwhile, cohesiveness is the positive ratio force during the second compression cycle to that of the first compression cycle, and express the extent to which the product can be deformed before it ruptures [31,46]. Cohesiveness is strictly correlated with the internal resistance of food structure and the property of a sample to stick to itself. It is preferable to have a high value in order to avoid the disintegration of the product during mastication. Gumminess is another texture parameter which is correlated with hardness and cohesiveness, being determined by hardness multiplied by cohesiveness [47].

A decrease of hardness was observed for the analyzed muffin samples made by addition of SP sourdough, giving to the final product SP PF 24 h a total value of 8.18 N. The hardness values obtained in samples with SP sourdough were statistically different from those obtained with OR sourdough. The same pattern was observed for cohesiveness; with increased values when SP 24 h sourdough was added, reaching a total value of 0.63.

Gumminess values for SP muffins ranged from 9.0 N to 4.76 N compared with OR muffins which values were between 9.21 N to 5.4 N and showing proportional patterns with hardness and chewiness. Chewiness is used to describe the level of difficulty needed in order to chew the food and to form the bolus before swallowing [31]. Regarding the SP PF and SP OR muffins, statistical analysis confirmed significant differences among samples showing an improvement in chewiness as the SP 24 h sourdough was added.

Springiness measures the elasticity and is the height that the food recovers during the time that elapses between the end of the first compression and the start of the second compression. It is an important mechanical characteristic that has been associated with the elastic and fresh aerated product by [48] and together with resilience could be used to describe crumb elasticity [49]. Resilience is the ratio of recoverable energy as the first compression is relieved, a lower value indicating that the muffins need more time to return to the initial form and chewiness (product of hardness × cohesiveness × springiness. N) [50]. In the present study, the springiness values increase as the muffins were manufactured with SP 24 h sourdough and similar pattern was observed with the resilience values.

The texture profile of the sample showed that sourdough could influence the features of the final baked products, by improving their texture. This result is also confirmed by Campo et al., (2016) [9] who found that the sourdough with *Lb. helveticus* had influenced the textural properties of the bread, improving the elasticity. This is in line with Rizzello et al., (2016) [51] which reported that sourdough with LAB could improve the hardness and the elasticity of the final baked goods. Also, Novotni et al., (2013) [52] confirmed that *Lactobaciluus plantarum* strain could be used for the manufacture of sourdough influencing the hardness, crumb firmness, and giving to the final product a superior texture characteriscs.

### 3.3. Aroma Volatile Compounds

The volatile compounds during sourdough fermentation could derive from several reactions like: (i) enzymatic oxidation or auto-oxidation of the lipids from the raw substrate with the development of aldehydes, ketones and 2-pentylfuran; (ii) microbial metabolism with formation of aroma compounds like 2–3 butanedione, acids and esters; or (iii) from genetic and environmental factors (terpenes), according to Aponte et al., (2013) [7].

The 21 aroma volatile derivatives from SP and OR sourdough identified in the present research were comprised of four alcohols, seven aldehydes, one ketone, five terpenes and terpenoids, three acids and other compounds like 2-pentylfuran—as listed in Table 6.

During rice flour fermentation with Lsp mainly aldehydes (hexanal, benzaldehyde, pentanal, nonanal, 2-methylbutanal, 3-methylbutanal, heptanal, pentanal), terpens and terpenoids (camphor, *p*-cymene, limonene, eucalyptol, carvone), alcohols (ethanol, 2–3 butanediol, pentan-1-ol, hexan-1-ol) and acids were formed (acetic, benzoic, hexanoic), as showed in Table 6.

Ethanol was the most representative alcohol between OR and SP sourdoughs during 24 h of fermentation and is a result of the alcoholic fermentation, derived from the pentose phosphate pathway of glucose, according to Lee et al., (2019) [17] or due to the heterofermentative LAB strains. The OR ethanol amount was significantly higher than in SP sourdough (*p* ≤ 0.05) due to the possible wild flour microbiota represented also by yeasts which lead to the formation of alcohols in a larger amount. This result is in agreement with [37].

Hexanal is considered by (Lee et al., 2019) [17] as an aroma compound resulted mainly from the enzymatic oxygenation of linoleic acid by lipoxygenase and hydroperoxide lyase. The hexanal amount could vary due to its oxidation to hexanoic acid through aldehyde dehydrogenase and due to its reduction to 1-hexanol during fermentation by alcohol dehydrogenase. Also, Lee et al., (2019) [17] reported that during fermentation of rice flour with different LAB strains, the combined amount of hexanal, 1-hexanol, and hexanoic acid was formed. Hexan-1-ol was identified in higher amount in SP 12 h sourdough, having a value of 11.44%, and decreased after 24 h of fermentation, having a final value of 6.09%.

Nonanal amount significantly decreased during SP fermentation with Lsp, because of Lsp possible activity on short-chain alcohol dehydrogenase with influence on the amount of this aroma compound. In the study reported by [17], nonanal was also identified in rice flour fermented with *Lactobacillus pentosus* and *Pediococcus lolii*. Also, [7] identified nonanal as the most abundant aldehyde during fermentation of chestnut and rice flours sourdough fermented with different LAB strains.

During SP 24 h fermentation, aldehydes like 2-methylbutanal and 3-methylbutanal were formed through isoleucine and leucine Strecker degradation. This result is in line to Lee et al., (2019) [17], who reported that during fermentation of rice with *Lactobacillus sakei* the two already mentioned aroma compounds were formed. Also, the acetophenone content significantly increased during the 24 h fermentation of SP, being statistically different from the OR content. A similar result was reported by Aponte et al., (2013) [7].

2-Pentylfuran, with an odor perception of green beans, buttery is the end product of 9-hydroperoxodes oxidation by lipoxygenase being found in many rice cultivars Lee et al., (2019) [17]. It was identified only in the SP sourdough after 12 h of fermentation and its amount decrease after 24 h of fermentation.

In the SP sourdough, from the terpenes and terpenoids group, the most representative were *p*-cymene and limonene which amount reached values of 12.3% and 15.01% respectively, followed by eucalyptol and carvone with values of 3.71 and 3.61% respectively. *p*-Cymene and limonene are responsible for the citrus, sweet, herbal, spicy and mint aroma notes, meanwhile eucalyptol and carvone are responsible for flavors like liquor, mint and pine flavors. Camphor belongs to the same group and gave a bitter taste to the sourdough. A larger amount of camphor was identified in OR sourdough. The presence of limonene and *p*-cymene in the sourdough could be justified by the presence of these volatile compounds in the rice flour [53].

The volatile compounds from the sourdough are different compared to the volatile compounds of the muffins mainly because of the sourdough volatile compounds, lipid oxidation, Maillard and caramelization reactions that occurs during baking.

In the SP and OR muffins, a total number of 18 compounds were identified as follows: two alcohols, six aldehydes, four ketones, two terpenes and terpenoids, two acids and two others compounds named 2-pentylfuran and dimethyl-disulfide, as listed in Table 7. The mainly alcohol from the final baked goods was ethanol, which was identified only in OR PF 12h muffins followed by hexan-1-ol which was identified in SP PF 24 h muffins. The most representative compounds from aldehydes group in SP PF muffins were 2-methylbutanal and 3-methylbutanal, being statistically different from the values obtained from OR PH muffins as showed in Table 7. 2-methylbutanal and 3-methylbutanal were formed during Strecker degradation of the alanine, leucine and isoleucine amino-acids, being the main aromatic outcomes, according to [54,55].

From the ketones group, only acetophenone was found in all muffin samples, reaching the highest value in SP PF 24 h and having a floral, almond odor. Limonene, the mainly compound from terpens and terpenoids group was found in SP PF 24 h, and its presence could be justified by the chemical compounds of rice and buckwheat, especially the carotenoids content could be correlated with limonene content [56]. 

In the OR PF baked products the main volatile compounds were represented by acetic acid, benzoic acid, dimethyl-disulfide, ethanol which gave an undesirable aroma to the final product such as vinegar, sour, garlic or acrid. This could be explained by the spontaneous fermentation of OR which was dominated by the microflora of the rice integral flour.

On the other hand, the presence of lactic acid in SP PH samples, support the idea that Lsp was responsible for the acidification sourdough during fermentation with influence on the sensorial profile of the sourdough due to the improving content on aroma compounds. This is in line with other researches, like [50,57].

### 3.4. Sensorial Analysis

**Figure 3:**
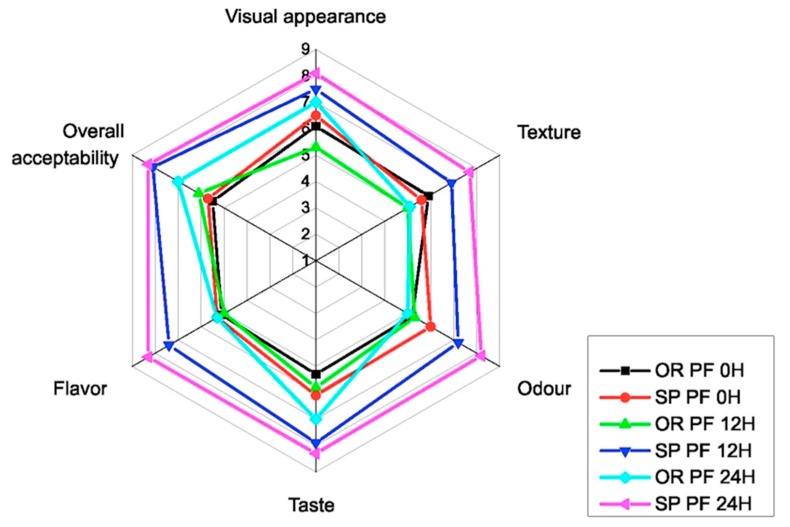
Hedonic scores for of GFM (gluten free muffins) obtained with inoculated (SP) and non-inoculated (OR) sourdoughs at different fermentation time: 0, 12, 24 h.

The panelists evaluated the SP PF 12 h and SP PF 24 h as having a good taste, flavor, and texture. The overall acceptability was 8.1 and 8.3 respectively for the aforementioned samples, which position the samples on the positive liking side of the hedonic scale, as showed in Figure 3. This could be explained by the presence of aroma volatile compounds such as 3-methylbutanal, 2-methylbutanal, acetophenone and limonene, which gave to the final baked product odors perception such as musty, fermented, mint. This result highlighted the fact that the sourdough is mainly used as an aroma improver for the bakery industry [37]. The idea that fermentation of gluten free flours by LAB could lead to the production of flavor compounds and generate specific aroma profiles and odorant compositions with influence on the quality aroma of the final baked goods was supported also by [8,13,58,59].

Also, the texture was accepted by the assessors as having the biggest total score of 7.7 for the SP PF 24 h, compared with the OR PF 24 h muffins which received a score of 5.1 which means neither like nor dislike. This is in line with other findings which support the idea that sourdough fermented with LAB could improve the texture of the final baked product [2,26,45,60].

## 4. Conclusions

*Lactobacillus spicheri* is a novel strain isolated from industrial rice sourdough but unexploited for the manufacturing of bakery products. To the best of our knowledge, a few published researches are available with this LAB strain in bakery products manufacturing. According to some authors, this LAB strain could be considered a probiotic strain which could be used in the production of functional foods. By this study a rice sourdough fermented with *Lactobacillus spicheri* DSM 15429 was used for gluten free muffins manufacturing in order to improve their textural and volatile profile. Results showed a significant improvement of textural parameters (hardness, cohesiveness, springiness, resilience) as well as of the volatile derivatives responsible for the aroma and the odor of the gluten free baked goods. Further studies will be conducted to emphasize the ability of the strain to produce bioactive compounds and to improve the nutritional value of the bakery products.

## Figures and Tables

**Table 1:** Proximate composition of rice integral flour.

Parameters	Proximate Composition * (%)
**Moisture**	10.4 ± 0.31
**Proteins**	8.5 ± 0.23
**Lipids**	2.8 ± 0.56
**Carbohydrates**	77.4 ± 1.31
**Ash**	0.9 ± 0.21

* Mean values of three different determinations followed by standard deviation.

**Table 2:** Samples codification and sampling times during sourdough fermentation with *Lactobacillus spicheri* DSM 15429 (Lsp) and without Lsp.

	Sample Codes	
	Sourdough with *Lactobacillus spicheri*	Sourdough without *Lactobacillus spicheri*
Sampling Times (h)	SP	OR
**0**	SP 0	OR 0
**4**	SP 4	OR 4
**8**	SP 8	OR 8
**12**	SP 12	OR 12
**24**	SP 24	OR 24

**Table 3:** Formulations for gluten-free muffins with sourdough fermented with *Lactobacilus spicheri* DSM 15429 strain and with spontaneously fermented sourdough (OR).

Ingredients (%)	SP Muffins	OR Muffins
**Treated rice flour**	32.50	32.50
**SP**	15.00	-
**OR**	-	15.00
**Inulin**	8.00	8.00
**Oatmeal**	10.00	10.00
**Corn starch**	7.00	7.00
**Eggs**	8.00	8.00
**Baking powder**	1.50	1.50
**Coconut butter**	5.00	5.00
**Buckwheat flour**	8.00	8.00
**Maple syrup**	5.00	5.00
**Total**	100.00	100.00

**Table 4:** Codification and sampling times during sourdough fermentation with Lsp (SP) and without Lsp (OR).

	Sample Codes	
	Muffins with *Lactobacillus spicheri* (SP)	Muffins without *Lactobacillus spicheri* (OR)
**Sampling Times (h)**	SP PF	OR PF
**0**	SP PF 0H	OR PF 0H
**12**	SP PF 12H	OR PF 12H
**24**	SP PF 24H	OR PF 24H

**Table 5:** Texture profile analysis of muffins sample *.

Muffins Samples	Hardness Cycle 1 [N]	Total Work Cycle 1 [mJ]	Hardness Cycle 2 [N]	Total Work Cycle 2 [mJ]	Resilience [mJ]	Springiness Index [n.a.]	Cohesiveness [n.a.]	Gumminess [N]	Chewiness [N]
OR PF 0H	23.93± 1.3 ^e^	63.56 ± 2.3^b^	27.06 ± 0.5 ^e^	34.36 ± 0.7 ^b^	0.19 ± 0.01 ^a^	0.78 ± 0.06 ^a^	0.38 ± 0.04 ^a^	9.21 ± 0.2 ^d^	4.84 ± 0.03 ^d^
SP PF 0H	24.78 ± 2.5 ^e^	67.03 ± 1.7 ^c^	29.62 ± 0.91 ^f^	47.40 ± 0.26 ^c^	0.19 ± 0.03 ^a^	0.75 ± 0.02 ^a^	0.36 ± 0.02 ^a^	9.00 ± 0.29 ^d^	4.79 ± 0.03 ^d^
OR PF 12H	17.74 ± 1.9 ^d^	95.85 ± 1.5 ^e^	18.27 ± 0.81 ^d^	27.00 ± 0.35 ^a^	0.20 ± 0.02 ^a^	0.70 ± 0.13 ^a^	0.41 ± 0.10 ^bc^	7.39 ± 0.65 ^c^	4.20 ± 0.07 ^c^
SP PF 12H	16.24 ± 1.09 ^c^	101.35 ± 1.2 ^f^	13.28 ± 0.7 ^c^	48.30 ± 0.2 ^c^	0.20 ± 0.01 ^a^	0.71 ± 0.01 ^a^	0.43 ± 0.01 ^a^	7.04 ± 0.16 ^c^	3.89 ± 0.02 ^b^
OR PF 24H	13.84 ± 0.3 ^b^	93.52 ± 0.7 ^d^	11.24 ± 0.43 ^b^	47.30 ± 0.31 ^c^	0.21 ± 0.01 ^a^	0.73 ± 0.02 ^a^	0.39 ± 0.02 ^ab^	5.40 ± 0.21 ^b^	4.00 ± 0.02 ^b^
SP PF 24H	9.52 ± 0.21 ^a^	59.72 ± 1.2 ^a^	8.18 ± 0.61 ^a^	26.35 ± 0.13 ^a^	0.33 ± 0.02 ^b^	0.81 ± 0.03 ^b^	0.50 ± 0.21 ^d^	4.76 ± 0.15 ^a^	3.17 ± 0.03 ^a^

Note: ^a–f^ different superscripts in a column indicate significant difference within samples (*p* < 0.05). * Each value was the mean of duplicate measurements. Muffins samples based on sourdough with *Lactobacillus spicheri* (SP) and without *Lactobacillus spicheri* (OR) at different sampling times 0, 12, 24 (h); n.a.—not applicable.

**Table 6:** Aroma compounds formed during 24 h of OR and SP sourdough fermentation *.

Volatile Compounds	Odor Perception	OR 0H	SP 0H	OR 12H	SP 12H	OR 24H	SP 24H
**Alcohols**							
2.3-Butanediol	Fruit, Creamy, Butter	N.D.	N.D.	6.40 ± 0.27 ^a^	N.D.	7.43 ± 0.03 ^b^	N.D.
Ethanol	Strong Alcohol, Ethereal, Medicinal	N.D.	N.D.	40.38 ± 0.06 ^b^	N.D.	8.31 ± 0.07 ^a^	N.D.
Hexan-1-ol	Ethereal, Oil, Alcohol, Green, Fruity, Sweet, Woody, Floral	N.D.	4.56 ± 0.23 ^a^	8.68 ± 0.56 ^c^	11.44 ± 0.34 ^d^	6.2 ± 0.07 ^b^	6.09 ± 0.78 ^b^
Pentan-1-ol	Balsamine, Oil, Sweet, Chemical Mint	N.D.	N.D.	N.D.	1.35 ± 0.78 ^a^	1.74 ± 0.55 ^a^	2.20 ± 0.22 ^b^
**Aldehydes**							
Benzaldehyde	Almond, String, Sharp, Sweet, Bitter, Cherry	3.98 ± 0.38 ^cd^	4.74 ± 0.31 ^d^	2.75 ± 0.51 ^b^	1.24 ± 0.03 ^a^	1.62 ± 0.27 ^a^	3.13 ± 0.11 ^bc^
3-Methylbutanal	Ethereal, Aldehydic, Chocolate, Peach, Fatty, Sour, Roasted Bread, Fruity, Fermented, Corn Flakes.	1.36 ± 0.12 ^a^	1.28 ± 0.22 ^a^	1.41 ± 0.33 ^b^	2.31 ± 0.29 ^c^	1.05 ± 0.18 ^c^	4.63 ± 0.23 ^d^
2-Methylbutanal	Musty, Cocoa, Coffee, Nut, Malty, Fruity, Sweet, Roasted.	N.D.	N.D.	N.D.	N.D.	1.28 ± 0.11 ^a^	5.06 ± 0.43 ^b^
Hexanal	Fresh, Green. Fatty, Aldehydic. Grass., Leafy, Fruity, Sweaty	44.54 ± 0.56 ^ef^	42.1 ± 0.37 ^e^	4.26 ± 0.76 ^a^	16.39 ± 0.55 ^d^	5.79 ± 0.23 ^b^	8.01 ± 0.88 ^c^
Pentanal	Almond, Malt, Pungent	5.91 ± 0.34 ^a^	6.17 ± 0.23 ^a^	N.D.	N.D.	N.D.	N.D.
Nonanal	Aldehydic, Rose, Waxy, Citrus. Orange, Floral	1.66 ± 0.12 ^c^	1.84 ± 0.34 ^c^	1.89 ± 0.56 ^cd^	0.51 ± 0.22 ^ab^	2.08 ± 0.31 ^e^	0.22 ± 0.17 ^a^
Heptanal	Fresh, Aldehydic, Fatty, Green.	N.D.	N.D.	0.27 ± 0.04 ^a^	N.D.	N.D.	N.D.
**Ketones**							
Acetophenone	Floral, Amond	8.97 ± 0.31 ^c^	8.89 ± 0.03 ^c^	5.39 ± 0.26 ^b^	10.31 ± 0.11 ^d^	2.40 ± 0.25 ^a^	12.10 ± 0.34 ^e^
**Terpenes and terpenoids**							
Camphor	Bitter taste	3.39 ± 0.12 ^b^	1.32 ± 0.10 ^ab^	1.55 ± 0.26 ^ab^	0.62 ± 0.29 ^a^	5.91 ± 0.63 ^c^	0.74 ± 0.20 ^a^
*p*-Cymene	Citrus, Sweet, Herbal, Spicy	2.61 ± 0.19 ^a^	3.16 ± 0.76 ^b^	1.91 ± 0.35 ^a^	5.31 ± 0.24 ^c^	1.37 ± 0.13 ^a^	12.3 ± 0.71 ^d^
Limonene	Citrus, Mint	10.24 ± 12 ^abc^	10.21 ± 0.21 ^c^	8.01 ± 0.76 ^b^	14.26 ± 0.24 ^cd^	6.51 ± 0.61 ^a^	15.01 ± 0.32 ^d^
Eucalyptol	Liquor, Mint. Pine.	2.69 ± 0.22 ^a^	1.98 ± 0.06	2.06 ± 0.34	1.76 ± 0.31 ^ab^	1.23 ± 0.51 ^b^	3.71 ± 0.24 ^c^
Carvone	Mint	2.15 ± 0.34 ^a^	2.03 ± 0.06 ^a^	1.52 ± 0.03 ^a^	3.94 ± 0.29 ^b^	0.68 ± 0.33 ^a^	3.61 ± 0.22 ^b^
**Acids**							
Acetic acid	Sharp, Acrid, Vinegar, Sour.	N.D.	N.D.	N.D.	25.19 ± 0.31 ^b^	41.75 ± 0.35 ^c^	19.13 ± 0.37 ^a^
Benzoic Acid	Faint Balsam, Urine.	12.51 ± 0.09 ^d^	9.22 ± 0.03 ^d^	12.55 ± 0.21 ^c^	5.74 ± 0.04 ^b^	5.65 ± 0.07 ^b^	0.30 ± 0.02 ^a^
Hexanoic acid	Sour, Fat, Sweat, Cheesy	N.D.	N.D.	N.D.	N.D.	N.D.	1.19 ± 0.05 ^c^
**Others**							
2-Pentylfuran	Green Beans, Butter, Metallic, Fruity	N.D.	N.D.	N.D.	2.64 ± 0.12 ^b^	N.D.	0.57 ± 0.03 ^a^

Note: ^a–f^ different superscripts in a row indicate significant difference within samples (*p* < 0.05). * Each value was the mean of duplicate measurements. Muffins samples based on sourdough with *Lactobacillus spicheri* (SP) and without *Lactobacillus spicheri* (OR) at different sampling times 0, 12, 24 (h); N.D.—not detected.

**Table 7:** Aroma compounds of the final baked muffins fermented with OR and SP sourdoughs at different fermentation times: 0 h, 12 h, 24 h *.

Volatile Compounds	Perceived Flavour	OR PF 0H	SP PF 0H	OR PF 12H	SP PF 12H	OR PF 24H	SP PF 24H
**Alcohols**							
Hexan-1-ol	Ethereal, Oil, Alcohol, Green, Fruity, Sweet, Woody, Floral	N.D.	N.D.	N.D.	N.D.	N.D.	2.32 ± 0.21
Ethanol	Strong, Alcohol, Ethereal, Medicinal.	N.D.	N.D.	7.01 ± 0.04	N.D.	N.D.	N.D.
**Aldehydes**							
Hexanal	Fresh, Green, Fatty, Aldehydic, Grass, Leafy, Fruity, Sweaty	20.73 ± 0.25 ^e^	22.8 ± 0.23 ^f^	7.39 ± 0.67 ^b^	5.44 ± 0.19^a^	11.87± 0.23 ^c^	13.45 ± 0.11 ^d^
Pentanal	Almond, Malt, Pungent	N.D.	N.D.	N.D.	N.D.	N.D.	2.81± 0.31
Benzaldehyde	Almond, String, Sharp, Sweet, Bitter, Cherry	N.D.	N.D.	1.48 ± 0.02 ^a^	5.98 ± 0.34 ^b^	1.3 ± 0.33 ^a^	7.4 ± 0.22 ^c^
3-Methylbutanal	Ethereal, Aldehydic, Chocolate, Peach, Fatty, Sour, Roasted Bread, Fruity, Fermented, Corn Flakes.	28.67 ± 0.11 ^a^	25.98 ± 0.27 ^a^	28.95 ± 0.54 ^b^	48.9 ± 0.02 ^d^	29.07 ± 0.24 ^b^	40.76 ± 0.45 ^c^
2-Methylbutanal	Musty, Cocoa, Coffee, Nut, Malty, Fruity, Sweet, Roasted.	13.56 ± 0.04 ^a^	14.88 ± 0.07 ^ab^	23.17± 0.56 ^e^	24.24 ± 0.76 ^e^	18.51 ± 0.89 ^d^	15.22 ± 0.45 ^c^
Nonanal	Aldehydic, Rose, Waxy, Citrus, Orange, Floral	N.D.	N.D.	0.39 ± 0.02 ^a^	N.D.	0.69 ± 0.03 ^a^	N.D.
**Ketones**							
2.3-Butanedione	Strong, butter, sweet, creamy, sour, caramel	N.D.	N.D.	N.D.	2.46 ± 0.03	N.D.	N.D.
2.3-Pentanedione	Acrid, Sweet, Butter, Caramel, Creamy, Nut	N.D.	N.D.	4.54 ± 0.02 ^a^	4.26 ± 0.01 ^a^	N.D.	N.D.
Acetophenone	Floral, Almond	1.08 ± 0.23 ^a^	0.97 ± 0.01 ^a^	2.13 ± 0.33 ^b^	3.08 ± 0.23 ^c^	2.23 ± 0.45 ^b^	4.71 ± 0.22 ^d^
2-Heptanone	Soapy, fruity, cinnamon	N.D.	N.D.	N.D.	N.D.	0.66 ± 0.21 ^a^	3.54 ± 0.32 ^b^
**Terpenes and terpenoids**							
Limonene	Citrus, mint	1.23 ± 0.18 ^a^	1.49 ± 0.34 ^a^	1.41 ± 0.52 ^a^	0.81 ± 0.23 ^a^	3.57 ± 0.47 ^b^	8.96 ± 0.05 ^c^
*p*-Cymene	Citrus, Sweet, Herbal, Spicy	N.D.	N.D.	N.D.	0.25^a^	N.D.	0.38 ^a^
**Acids**							
Benzoic acid	Faint, Balsam, Urine	3.38 ± 0.45 ^ab^	4.9 ± 0.03^b^	1.54 ± 0.02 ^a^	2.19 ± 0.44 ^a^	1.93 ^a^	N.D.
Acetic acid	Sharp, Acrid, Vinegar, Sour.	25.32 ± 0.75 ^d^	23.09 ± 0.67 ^c^	15.22 ± 0.23 ^a^	N.D.	18.30 ± 0.01 ^b^	N.D.
**Others**							
2-pentylfuran	Green beans, Butter, Metallic, Fruity	N.D.	N.D.	0.72 ± 0.01 ^a^	N.D.	2.0 ± 0.33 ^b^	N.D.
Dimethyl-disulfide	Garlic. unpleasant	6.03 ± 0.33 ^a^	05.89 ± 0.22 ^a^	6.07 ± 0.11 ^b^	2.39 ± 0.29 ^a^	9.87 ± 0.08 ^b^	0.45 ± 0.01 ^a^

Note: ^a^^–f^ different superscripts in a row indicate significant difference within samples (*p* < 0.05). * Each value was the mean of duplicate measurements. Muffins samples based on sourdough with *Lactobacillus spicheri* (SP) and without *Lactobacillus spicheri* (OR) at different sampling times 0, 12, 24 (h); N.D.—not detected.
